# Lifetime risk of solid tumors and leukemia in Down Syndrome: a population-based Swedish matched cohort study

**DOI:** 10.1038/s41416-025-03318-5

**Published:** 2025-12-26

**Authors:** Alexandra Wachtmeister, Benedicte Bang, Ida Nordgren, Anna Martling, Bertil Johansson, Yunxia Lu, Anna Skarin Nordenvall, Giorgio Tettamanti, Ann Nordgren

**Affiliations:** 1 Department of Molecular Medicine and Surgery, Karolinska Institutet, Stockholm, Sweden; 2 Department of Pelvic Cancer, GI Oncology and Colorectal Surgery Unit, Karolinska University Hospital, Stockholm, Sweden; 3 Department of Clinical Genetics, Pathology, and Molecular Diagnostics, Laboratory Medicine, Region Skåne, Lund, Sweden; 4 Department of Population Health and Disease Prevention & Department of Epidemiology and Biostatistics, Joe C. Wen School of Population & Public Health, University of California, Irvine, CA USA; 5 Department of Radiology, Karolinska University Hospital, Stockholm, Sweden; 6 Unit of Epidemiology, Institute of Environmental Medicine, Karolinska Institutet, Stockholm, Sweden; 7 Department of Clinical Genetics and Genomics, Karolinska University Laboratory, Karolinska University Hospital, Stockholm, Sweden; 8 Department of Clinical Genetics and Genomics, Sahlgrenska University Hospital, Gothenburg, Sweden

**Keywords:** Cancer epidemiology, Cancer genetics, Paediatric cancer, Cancer epidemiology

## Abstract

**Background:**

Individuals with Down syndrome have an elevated risk of childhood leukaemia and are suggested to have a reduced risk of solid tumours in adulthood. However, it remains unclear which cancer subtypes contribute to this pattern and the lifetime cancer risk.

**Methods:**

This Swedish population-based matched cohort study investigated age- and subtype-specific cancer risks in Down syndrome. National healthcare registers were used to include 9742 individuals with Down syndrome, born in Sweden between 1930–2017. Each individual was matched by birth year, sex, and birth county to 50 comparisons. Hazard ratios (HRs) and 95% confidence intervals (95% CI) were calculated using Cox proportional hazard models.

**Results:**

Children with Down syndrome had a 20-fold increased risk of acute lymphoblastic leukaemia (ALL), and nearly a 500-fold increased risk of acute myeloid leukaemia (AML) before the age of 5. In contrast, individuals with Down syndrome had an overall lower risk of solid tumours, with significantly decreased risks for breast, prostate, lung, colorectal, gynaecological cancers, and melanoma, in adults. However, an increased risk was observed for testicular cancer and chondrosarcoma/chondroblastoma.

**Conclusion:**

We present the most comprehensive profile of cancer risk in Down syndrome, aiming to guide clinical practices, encourage tailored surveillance recommendations, and incite research on chromosome 21’s role in oncogenesis.

## Introduction

Down syndrome is caused by extra genetic material from either the entire or a critical part of chromosome 21. Down syndrome is the most common chromosomal aberration, occurring in approximately 1 in 800 live births. Down syndrome is characterised by intellectual disability, dysmorphic features, malformations, and growth retardation [1]. Advances in medical care, especially in monitoring and treatment of congenital heart defects, have significantly improved life expectancy, allowing more individuals with Down syndrome to reach adulthood and elderdom [2].

Throughout life, individuals with Down syndrome display a unique composition of cancer risk. During childhood, they have an increased risk of developing acute leukaemia, with the risk of acute myeloid leukaemia (AML) increased 150–400 times in children under 5 years and the risk of acute lymphoblastic leukaemia (ALL) increased 20–30 times during childhood [3–6].

In contrast to leukaemia risk, previous studies have reported an overall decreased risk for solid tumours; however, it is not fully known which tumour types contribute to this reduced risk [7, 8]. A lower risk for breast, lung, and cervical cancers has been shown in previous studies [8–10]. Other studies have reported a lower risk of endocrine, prostate, skin, and gastrointestinal cancers, although results between the studies are contradictory [8, 9, 11, 12]. A 2- to 5-fold increased risk has been shown for testicular cancer and an elevated risk has been suggested for stomach, liver, and gallbladder cancer [8, 9, 12, 13].

It is essential to attain information on age-dependent and long-term risk of leukaemia and subtypes of other tumours to inform clinical practices and develop effective strategies for screening, early detection, and treatment. Additionally, understanding the cancer profile in individuals with Down syndrome could enable future insights on oncogenesis, for both individuals with Down syndrome and others. Using nationwide Swedish high-quality register data, we aim to bring novel insights and investigate cancer risk for both haematological malignancies and solid tumours throughout the life of individuals with Down syndrome.

## Materials and methods

### Study design

In this population-based matched cohort study, using data from Swedish national demographic and healthcare registers, we investigated cancer risk in individuals with Down syndrome. Data from the registers were linked using the unique personal identity number assigned to all Swedish residents [14]. The study was conducted according to the STROBE reporting guidelines. We included individuals with Down syndrome born in Sweden between 1^st^ of January 1930 and 31^st^ of December 2017, and randomly selected 50 comparisons without Down syndrome from the Swedish Total Population Register [14] using sex, birth year, and county of birth as matching factors. Ethical approval was granted by the Stockholm Regional Ethics Committee, which waived the need for informed consent from study participants.

### Identification of individuals with Down syndrome

We used the Medical Birth Register (MBR) and the National Patient Register (NPR), held by the Swedish National Board of Health and Welfare, to identify individuals with at least one diagnosis of Down syndrome born 1930–2017 (*n* = 10,995). Both NPR and MBR record diagnoses according to the International Classification of Diseases (ICD) (see Table S1 for the ICD-codes used), and the registers are continuously updated. MBR includes information on birth characteristics, congenital malformations, and perinatal diagnoses for all children born in Sweden since 1973 [14]. NPR includes information on inpatient care since 1964, reaching nationwide coverage in 1987. Initially, only information on somatic care was included in the register. By 1973, inpatient data were reported from 18 of the 26 counties in Sweden, including the largest ones [15]. Data on outpatient specialist visits have been available since 2001 [14].

We excluded individuals with Down syndrome born outside of Sweden as well as those lacking data on the matching factors sex, birth year, and county of birth (*n* = 1120; see flow chart in Fig. S1). To prevent misclassification of individuals who may have been incorrectly presumed to have Down syndrome before accurate testing could be conducted, we excluded individuals who were assigned a diagnostic code for another congenital syndrome after their most recent Down syndrome diagnosis (*n* = 128). Among the remaining cohort, 5 of 208 individuals with available clinical genetic data (patients diagnosed at Karolinska University Hospital in Stockholm 1993–2017) had results indicating a different diagnosis and were therefore excluded.

### Identification of cancer cases

Information regarding cancer diagnoses was retrieved from the National Cancer Registry (NCR). Since 1958, it has been compulsory in Sweden to report all malignant tumours, leukaemias, and certain premalignant and benign tumours to NCR together with the corresponding ICD/ICD-O codes. NCR has a high overall completeness and quality [14]. Cancer cases were identified and classified according to the topography and morphology coding used in NCR. Only cancers classified as malignant in the NCR were included in the study, except for leukaemias (ICD-8 204-207) and CNS tumours (excluding meningiomas) that were always considered malignant. Diagnoses before age 20 were considered childhood cancer, and only the first cancer diagnosis was included.

In analyses for childhood cancer, we restricted our cohort to individuals born after 1958 (as NCR was established in 1958) to minimize outcome misclassification (*n* = 7631).

### Statistical analysis

Descriptive statistics were used to describe the study participants’ characteristics. Cox proportional hazard models were used to estimate the associations between Down syndrome and cancer, with results presented as hazard ratios (HRs) and 95% confidence intervals (CI). All individuals were followed until death, emigration, first cancer diagnosis, or end of follow-up (2017-12-31). We tested proportional hazard assumption using Schoenfeld’s residuals and non-proportionality was indicated by an asterisk in the tables. All analyses were inherently adjusted for sex, birth year, and birth county. Childhood cancer analyses were also adjusted for maternal and paternal age at delivery ( < 25, 25 to 34, >34 years), and highest level of parental education (primary, secondary, postsecondary). Data on covariates were retrieved from the Multi-Generation Register, the Total Population Register and the Longitudinal Integration Database for Health Insurance and Labour Market Studies (from 1990 and onward), all held by Statistics Sweden [14]. To evaluate the impact of the shorter life expectancy in individuals with Down syndrome on solid tumour risk, we performed a post-hoc analysis restricting the age of cancer diagnosis between 20 and 60 years. For the common cancer sites lung and prostate, individuals were included from age 40 (instead of 20) as these cancers mainly occur in older individuals. Additionally, we conducted a sensitivity analysis including only individuals with two or more Down syndrome diagnoses (*n* = 8361). Furthermore, we conducted a sensitivity analysis investigating childhood cancer risk among individuals born between 1973 and 2017 (*n* = 5977), when the NPR coverage was higher and the MBR had been established. We also investigated adult cancer risk in individuals born between 1950 and 2017 (*n* = 8457), when registry coverage was higher, and between 1938–2017 (*n* = 9473), ensuring that the NCR had been established by the time all individuals reached age 20. Lastly, we did a sensitivity analysis where we stratified testicular cancer risk by cryptorchidism. The absolute risk of childhood leukaemia was estimated during the period 1973-2017. SAS 9.4 software was used for data preparation and Stata 16.1 for statistical analyses.

## Results

### Study population’s characteristics

Our cohort comprised 9742 individuals diagnosed with Down syndrome and 487,100 matched comparisons born in Sweden 1930–2017. Among the individuals with Down syndrome, the majority were male (54.2% vs. 45.8%). As expected, advanced maternal age ( ≥ 35 years at delivery) was more common in the Down syndrome (39.9%) than among the matched comparisons (14.0%). Also, the proportion of fathers ≥35 years at delivery was higher in Down syndrome than in the comparison group (46.1% vs. 26.5%) (Table 1).

**Table 1:** Characteristics at baseline and end of follow-up for all study participants.

	Down syndrome	Matched comparisons
	No (%)	No (%)
Total number of individuals	9742 (100%)	487,100 (100%)
Sex		
Male	5282 (54.2%)	264,100 (54.2%)
Female	4460 (45.8%)	223,000 (45.8%)
Birth year		
1930–1949	1285 (13.2%)	64,350 (13.2%)
1950–1969	2122 (21.8%)	106,100 (21.8%)
1970–1989	2532 (26%)	126,600 (26%)
1990–2009	2859 (29.3%)	142,950 (29.3%)
2010–2017	944 (9.7%)	47,200 (9.7%)
Maternal age at delivery		
<25	1330 (13.7%)	129,364 (26.6%)
25–34	4177 (42.9%)	277,391 (57%)
≥35	3889 (39.9%)	68,135 (14%)
Missing data	346 (3.6%)	12,210 (2.5%)
Mean age (SD)	33.0 (6.7)	28.7 (5.6)
Paternal age at delivery		
<25	630 (6.5%)	62,401 (12.8%)
25–34	3972 (40.8%)	270,245 (55.5%)
≥35	4486 (46.1%)	129,156 (26.5%)
Missing data	654 (6.7%)	25,298 (5.2%)
Mean age (SD)	35.5 (7.6)	31.8 (6.4)
Highest parental education		
Primary	1628 (16.7%)	81,887 (16.8%)
Secondary	3175 (32.6%)	181,187 (37.2%)
Postsecondary	3680 (37.8%)	185,337 (38%)
Missing data	1259 (12.9%)	38,589 (7.9%)
Cryptorchidism		
Diagnosis >6 months of age	307 (5.8%^a^)	3438 (1.3%^a^)
Age at end of follow-up		
<10	1986 (20.4%)	73,550 (15.1%)
10–19	1351 (13.9%)	69,003 (14.7%)
20–29	1360 (14%)	83,189 (17.1%)
30–39	1129 (11.6%)	63,883 (13.1%)
40–49	1192 (12.2%)	58,954 (12.1%)
50–59	1573 (16.2%)	53,972 (11.1%)
60–69	995 (10.2%)	48,528 (10%)
70–79	146 (1.5%)	29,864 (6.1%)
80+	10 (0.1%)	6157 (1.3%)
Reasons for end of follow-up		
Cancer diagnosis	312 (3.2%)	27,025 (5.6%)
Median age at diagnosis (IQR)	21.76 (46.4)	59.1 (19.5)
Migration	146 (1.5%)	35,278 (7.2%)
Median age at migration (IQR)	5.9 (6.5)	21.9 (23.9)
Death	3180 (32.6%)	30,130 (6.2%)
Median age at death (IQR)	53.9 (36)	61.2 (28.4)
Dec 31, 2017	6489 (66.6%)	400,628 (82.2%)
Median age at end follow-up (IQR)	26.8 (30.2)	31.7 (36)

Sex given at birth.

^a^Procent (%) of male individuals.

The Down syndrome cohort included 316,233 person-years at risk and 25,368 person-years at risk over age 50. In relation to the matched comparisons, more individuals with Down syndrome ended their follow-up <10 years (20.4% vs. 15.1%) and fewer ended follow-up above 70 years (70–79 (1.5% vs. 6.1%) and 80+ (0.1% vs. 1.3%)). Apart from that, the Down syndrome and matched comparison groups had similar age and person-years distribution categories over time (Table 1). With regards to mortality, 32.6% of individuals with Down syndrome died before end of follow-up (Dec 31, 2017), whereas only 6.2% died in the matched comparison group (Table 1).

### Distribution of cancer cases and incidence rates

**Fig. 1:**
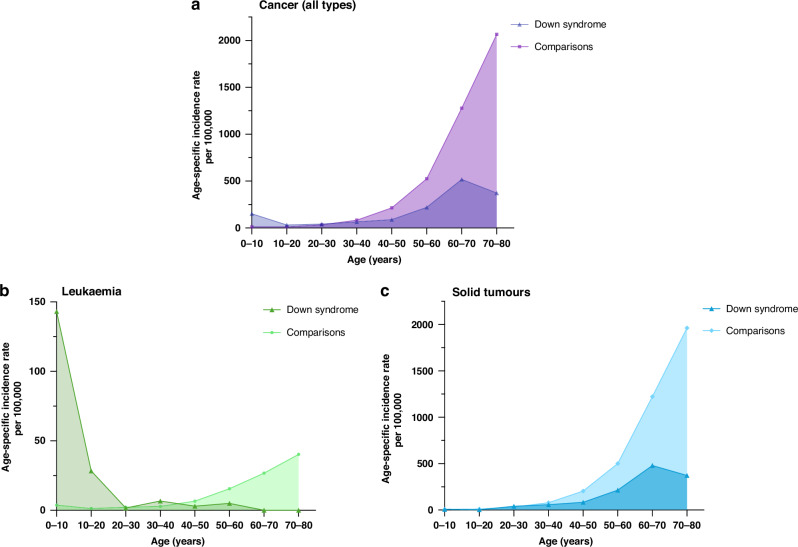
Incidence rates of cancer in individuals with Down syndrome and comparisons. Incidence rates for (**a**) all cancer types, (**b**) leukaemia, and (**c**) solid tumours (including lymphomas).

Cancer diagnoses were less common among individuals with Down syndrome (312 cancer cases, 3.2% vs. 27,025 cancer cases, 5.6%) and the cancer age distribution showed a different pattern in individuals with Down syndrome than in comparisons. Incidence rates of cancer were higher in individuals with Down syndrome during childhood and after that lower throughout life (Fig. 1a). The pattern was supported by high leukaemia incidence rates early in life (Fig. 1b) and low incidence rates for solid tumours throughout life (Fig. 1c).

### Risk of solid tumours

The risk of solid tumours was lower in adult individuals with Down syndrome than comparisons (HR 0.52, 95% CI 0.44–0.60); the same trend was seen in all ages above 40 (age groups 40–59, 60 + ). For childhood cancer the risk estimates were non-significant (Table 2).

**Table 2:** Risk of solid tumours (including lymphomas) in individuals with Down syndrome, stratified by age, sex, and site of cancer.

	Down syndrome	Comparisons	Hazard ratio
	No.	No.	(95% CI)^a^
**Adult solid tumours (20 + y)**	148	24 099	0.51^b^ (0.43–0.60)
Age at diagnosis			
20–39	49	28 13	0.93 (0.70–1.23)
40–59	70	9345	0.42 (0.33–0.53)
60+	29	11,941	0.40 (0.28–0.57)
Sex			
Female	76	12,189	0.48 (0.38–0.60)
Male	72	11,910	0.54 (0.43–0.69)
**Childhood solid tumours (0–19 y)**	10	723	0.73 (0.39–1.36)
Age at diagnosis			
<5	5	239	1.03 (0.42–2.51)
5–19	5	484	0.57^b^ (0.23–1.37)
Sex			
Female	3	336	0.47 (0.15–1.46)
Male	7	387	0.96 (0.45–2.03)

In the analyses of adult cancer, individuals were included from 1930–2017. For the childhood cancer analyses (cancer <20 years), only individuals born 1958–2017 were included.

^a^Hazard ratios (HRs) were inherently adjusted for matching factors (birth year, sex and birth county) in all analyses. For childhood cancer cases (cancer <20 years), HRs were also adjusted for maternal age, paternal age, and highest educational level.

Sex given at birth

^b^Does not meet proportional hazard assumptions.

### Site-specific risk of solid tumours

**Fig. 2:**
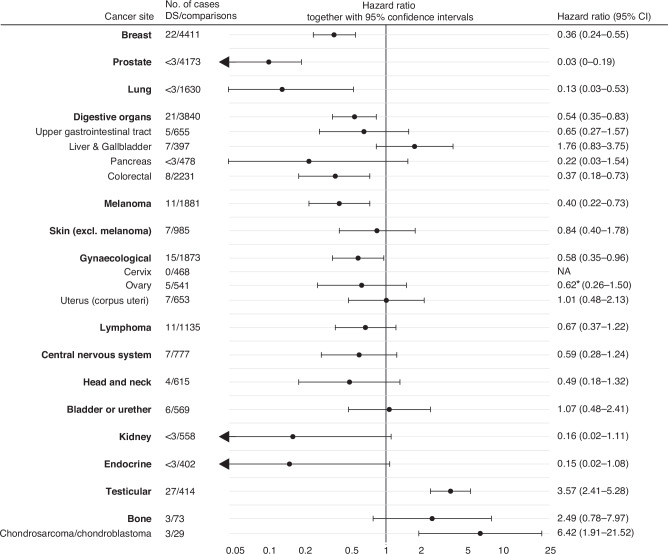
Risk of solid tumours in adult individuals with Down Syndrome, stratified by tumour site. Upper gastrointestinal tract includes esophagus, stomach, and small intestine. Gynaecological site includes vulva, vagina, cervix uteri, ovary, uterus and other unspecified female genitalia. Hazard ratios were inherently adjusted for matching factors (birth year, sex, and birth county) in all analyses. For the cancer sites lung and prostate, individuals were included from age 40 (instead of 20) as these cancers mainly occur in older individuals. NA = not applicable.

In the analyses stratified by cancer site, the tumour risk in Down syndrome was generally lower than comparisons across most sites, except for testicular cancer (HR 3.49, 95% CI 2.36–5.17), chondrosarcoma/chondroblastoma (HR 6.42, 95% CI 1.91–21.52), and for liver and gallbladder cancers (HR 1.71, 95% CI 0.83–3.64) (Fig. 2). Significantly decreased risks were found for breast (HR 0.36, 95% CI 0.24–0.55), prostate (HR 0.03, 95% CI 0–0.19), lung (HR 0.14, 95% CI 0.04–0.57), colorectal (HR 0.37, 95% CI 0.18–0.73), gynaecological (HR 0.58, 95% CI 0.35–0.96) cancers and melanoma (HR 0.40, 95% CI 0.22–0.73). The remaining sites; kidney, endocrine, pancreas, head and neck, ovarian, upper gastrointestinal tract (esophagus, stomach, and small intestine), CNS tumours, and lymphomas, all showed risk estimates trending toward a decreased risk, although not statistically significant (Fig. 2).

All testicular cancer cases in individuals with Down syndrome were diagnosed between age 17–55 (mean age 32.1, SD ± 9.7). A 3-fold increased risk of testicular cancer was observed in individuals with Down syndrome without cryptorchidism (HR 3.41, 95% CI 2.29–5.08) compared to comparisons, but an even higher association was found in individuals with Down syndrome and cryptorchidism (9.47, 95% CI 2.74–32.73) (Table 3). Stratified by morphology, the testicular cancer risk was increased for both seminomas and non-seminomas, although higher for seminomas (Table 3).

**Table 3:** Risk of testicular cancer in individuals with Down Syndrome, stratified by prior cryptorchidism, morphology, and age at diagnosis.

	Down syndrome	Comparisons	Hazard ratio
	No.	No.	(95% CI)^a^
**All testicular cancer**	29	439	3.61 (2.47–5.26)
**Stratified by cryptorchidism**			
Comparisons without cryptorchidism^b^	-	429	1(ref)
Comparisons with cryptorchidism^b^	-	10	2.09 (1.1–3.97)
DS without cryptorchidism^b^	26	-	3.41 (2.29–5.08)
DS with cryptorchidism^b^	3	-	9.42 (2.72–32.55)
**Morphology**			
Seminoma	16	154	5.80 (3.45–9.77)
Non-seminoma	8	157	2.95 (1.44–6.03)
Teratoma	<3	57	2.06 (0.50–8.47)
Embryonal carcinoma	4	45	4.81 (1.71–13.53)
**Age at diagnosis**			
<5	0	3	-
5–19	<3	21	4.41 (0.96–20.23)
20–39	23	323	3.82 (2.49–5.84)
40–59	4	81	2.77 (1.01–7.61)
60+	0	10	-

In the analyses for adult cancer, individuals were included from 1930–2017. For the childhood cancer analyses (cancer<20) only individuals born 1958–2017 were included.

^a^Hazard ratios were inherently adjusted for matching factors (birth year, sex and birth county) in all analyses. For the childhood cancer cases (cancer <20 years), hazard ratios were also adjusted for maternal age, paternal age, and highest educational level.

^b^Cryptorchidism was defined as a diagnosis of retentio/cryptorchidism after 6 months of age.

Our findings from the main analysis were supported by the post-hoc analyses restricting our cohort to <60 years of age (Table S3). In the sensitivity analysis excluding individuals with only one Down syndrome diagnosis, the overall same associations were found as in the main analysis. However, the risk for solid tumours ages 0–5 was lower and for CNS tumours in adults the risk was significantly reduced. Additionally, the suggestion of an increased risk of liver and gallbladder cancer was no longer evident and there were no cases of lung cancers among Down syndrome individuals (Table S4).

### Leukaemia risk

**Fig. 3:**
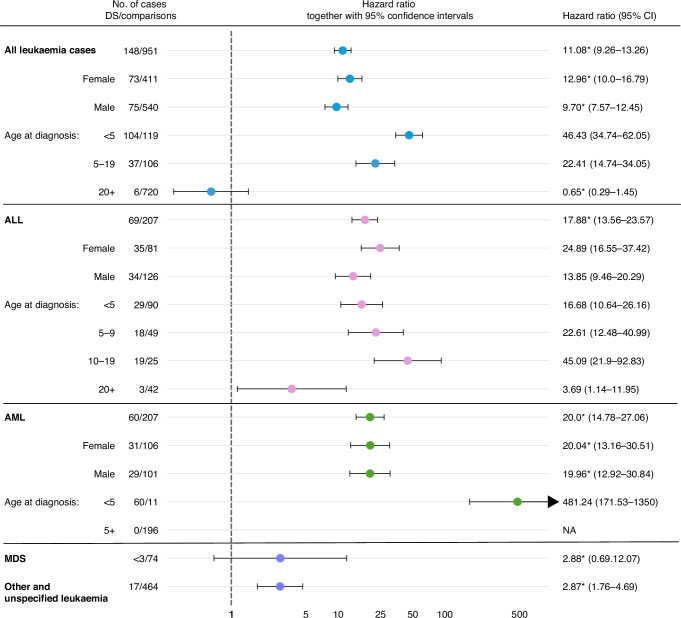
Leukaemia risk in individuals with Down syndrome, stratified by age, sex and leukaemia subtype. In the analyses for adult cancer, individuals were included from 1930–2017. For the childhood cancer analyses (cancer at age <20) only individuals born 1958–2017 were included. Hazard ratios were inherently adjusted for matching factors (birth year, sex, and birth county) in all analyses. For the childhood cancer cases (cancer <20 years), hazard ratios were also adjusted for maternal age, paternal age and highest educational level. Sex was defined as the sex given at birth. ALL acute lymphoblastic leukaemia, AML acute myeloid leukaemia, AMKL acute megakaryoblastic leukaemia, MDS myelodysplastic syndrome, NA not applicable. *Does not meet proportional hazard assumptions.

Acute lymphoblastic leukaemia (ALL) was the most common subtype of leukaemia in individuals with Down syndrome (*n* = 69). Individuals with Down syndrome had more than a 20-fold increased risk of ALL during childhood (HR 21.86, 95% CI, 16.06–29.77). In relation to comparisons, the risk of ALL peaked at age 10–19 (HR 45.09, 95% CI 21.9–92.83), but was high during early childhood as well. For acute myeloid leukaemia (AML) in Down syndrome, all cases were diagnosed before 5 years of age, which corresponded to an HR of 481.24 (95% CI 171.53–1350) during the first five years of life and an HR of 472.81 (95% CI 64.56–3462) for children <1 year (Fig. 3 and Table S2). Out of 60 AML cases in Down syndrome, 13 were characterised as acute megakaryoblastic leukaemia (AMKL), whereas only one of the comparisons was AMKL.

**Fig. 4:**
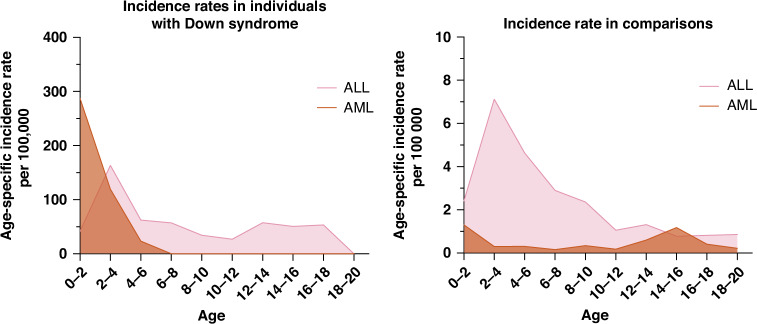
Incidence rates of AML and ALL in individuals with Down syndrome and comparisons. ALL acute lymphoblastic leukaemia, AML acute myeloid leukaemia.

In individuals with Down syndrome, the incidence rates for AML peaked during the first two years of life. For ALL, incidence rates peaked around 2–4 years, but remained elevated throughout childhood and adolescence, contrasting to the pattern seen in comparisons (Fig. 4).

Individuals with Down syndrome had an absolute risk of 2.49% for developing leukaemia during childhood, compared to 0.07% in matched comparisons.

In the sensitivity analysis investigating childhood cancer risk among individuals born between 1973 and 2017 the results were consistent with those of the main analysis (Table S5). Likewise, in sensitivity analysis investigating adult cancer risk among individuals born 1950–2017 and 1938–2017 the results showed the same association as in the main analysis (Table S6 and Table S7).

## Discussion

In this study, comprising 9742 individuals with Down syndrome born in Sweden between 1930 and 2017, we found that adult individuals with Down syndrome had an overall decreased risk of solid tumours. For the most prevalent cancer sites—breast, lung, colorectal, and prostate - individuals with Down syndrome demonstrated less than half the risk of their matched comparisons. Furthermore, a reduced risk was observed for several other cancer sites. In contrast, an increased risk was found for testicular cancer, chondrosarcoma/chondroblastoma, and suggested for liver and gallbladder cancer. As expected, ALL and AML risk was substantially increased among children with Down syndrome.

Our findings of an increased risk of childhood ALL and AML are consistent with previous studies [4–6]. The pattern of ALL risk observed in our study, with a peak in hazard ratio at age 10–19 and higher incidence rates than comparisons during adolescence, suggests that, apart from the common age peak between 2 and 4 years, later onset of ALL is more common in individuals with Down syndrome. Two large studies investigating outcomes of ALL also suggest that ALL in Down syndrome is more common in children over 10 years [16, 17]. Furthermore, our study suggests a higher risk of ALL in females with Down syndrome. This has been suggested by some previous studies, but results have been contradictory [9, 16, 17].

An increased risk of testicular cancer in individuals with Down syndrome has been repeatedly reported, and our results confirm this association [8, 9, 12, 13]. The increased risk has been suggested to result from the higher incidence of cryptorchidism in individuals with Down syndrome [9, 18]. However, our stratified analyses indicated a slightly lower, yet still elevated, risk of testicular cancer in individuals with Down syndrome without cryptorchidism. These findings suggest that while cryptorchidism is a risk factor, it does not fully explain the increased risk of testicular cancer in Down syndrome.

Some previous studies and case reports have suggested increased risk of liver and gallbladder cancer, which is also indicated by the results in our main analysis (HR 1.76, 95% CI 0.83–3.75) [9, 12, 19]. Contributing risk factors to this possible association could include the higher frequence of cholelithiasis, gallbladder disease [20], and obesity [7] in Down syndrome.

Lastly, our study shows an increased risk of chondrosarcoma/chondroblastoma of the bone. Previous evidence of an association between bone tumours and Down syndrome is sparse, suggested only by a few case reports and a single case in a cohort study, and subsequently needs further investigation [9, 21, 22].

The risk of solid tumours was decreased in individuals with Down syndrome, consistent with previous reports [7, 8]. Studies investigating site-specific tumour incidence often lack power due to small cohort sizes and limited follow-up times. However, our findings of lower risks for breast, cervical/gynaecological, and lung cancers are all supported by statistically significant findings in at least two previous studies [8–10], and for melanoma in one previous study [8]. We also found significantly lower risks for colorectal and prostate cancers, which have been proposed in some earlier reports, albeit with varying consistency and evidence levels [8, 9, 12]. For instance, the risk of colorectal cancer has been reported as lower, same, and higher in individuals with Down syndrome [8, 9, 11]. Additionally, our study indicate a lower risk of various other solid tumours, a finding supported by the largest study on mortality in individuals with Down syndrome, which included 18 000 individuals in the US and reported substantially lower mortality for all solid tumours except testicular cancer [23].

The reason for this protective association of a broad spectrum of cancers in individuals with Down syndrome is still not known. Certainly, lifestyle and risk behaviours in individuals with Down syndrome are likely to play a role. For instance, risk factors such as smoking, sun-exposure, and sexual activity, which might be less frequent in individuals with Down syndrome, are most likely contributing to the decreased risk of lung cancer, melanoma, and cervical cancers, respectively. However, other risk factor associations are more conflicting. For instance, obesity, which is more common in individuals with Down syndrome [7], is associated with an increased risk of breast and colorectal cancer [24], contradicting our findings. If lifestyle behaviours associated with intellectual disability were a key factor to the cancer profile in Down syndrome, then a similar pattern of cancer risks would be expected in other individuals with intellectual disability. However, this pattern is not observed [25, 26]. The unique cancer distribution in Down syndrome strengthens the proposition that an excess of genes located on chromosome 21 plays an essential role in their cancer predisposition pattern.

Knowledge about the potential underlying genetic mechanisms behind the protective association between trisomy 21 and solid tumours is limited. Studies have identified an anti-angiogenetic effect of the *DSCR1* and *DYRK1A* genes on chromosome 21, which are hypothesised to have a tumour-suppressing effect [27]. In addition, other cancer-associated genes, such as *ETS2*, are located on chromosome 21, and could possibly play a role in the cancer susceptibility in Down syndrome [28]. Interestingly, and in contrast to haematological malignancies, it has been suggested that chromosome 21 is more commonly lost in solid tumours in general, which further supports a genetic underlying cause to the decreased risk of solid tumours seen in this study [29, 30].

### Strength and limitations

To our knowledge, this is the largest study with the longest follow-up period investigating cancer incidence in individuals with Down syndrome. As our study includes almost 10,000 individuals with Down syndrome, it is 2–4 times larger than comparable previously published studies and includes substantially more person-years above 50 years of age. Another strength is the use of Swedish nationwide and high-quality registry data.

Despite this, we would ideally have included more person-years at a higher age to fully assess lifetime cancer risk. Naturally, this is limited due to the historically shorter life expectancy in individuals with Down syndrome, which is improving fast with modern medicine. As some cancers, such as prostate and colorectal cancers, mainly occur in older ages, we wanted to evaluate how the decreased cancer risks observed in this study were affected by the shorter life expectancy in the Down syndrome population. To test our results for this, we conducted a sensitivity analysis only including individuals aged 40–60. We used 60 years as cut-off since the Down syndrome cohort had an age and person-years distribution comparable to the matched comparisons until 60 years of age. The analysis showed the same protective patterns as in the main analysis, strengthening our main findings (Table S3).

The identification of individuals with Down syndrome was done through diagnostic codes and not confirmed by review of hospital records (except in 208 cases with available genetic data). However, if individuals without Down syndrome were wrongly included in our exposed cohort, we believe this would lead to an underestimation of our findings. To evaluate exposure misclassification, we did a sensitivity analysis where only individuals with two or more Down syndrome diagnoses were included. The results from this analysis showed some non-significant differences; however, the overall significant associations were the same as in the main analysis (Table S4).

Lastly, some cancer subgroups were limited by ICD/ICD-O coding classifications. For example, chondroblastoma and chondrosarcoma were grouped together as they share the same code in ICD-7, despite being clinically distinct entities.

### Clinical implications and future studies

Define the cancer risk profile in individuals with Down syndrome is essential to enable informed decision-making regarding clinical surveillance regimens. The results presented in this study compile the most comprehensive and detailed cancer profile to date. In the review of Rethoré et al. on surveillance recommendations for individuals with Down syndrome, they suggest withholding from systematic mammography screening of breast cancer and implementing annual palpation by health professionals after 50 years of age [31], a view also supported by others [32]. In addition, they propose inclusion in cervical cancer screening programs only if sexually active. Given that our results strengthen earlier findings, we propose that altered and tailored surveillance regimens in individuals with Down syndrome should be considered. As our study also reports a substantially decreased risk of colorectal cancer, evaluation of appropriate surveillance regimens for colorectal cancer, especially regarding invasive surveillance procedures like colonoscopy, should be made. Even if the potential harm and burden of cancer screening in this population must be assessed, especially for invasive and “sensitive” testing, it is also of utmost importance to consider this group’s vulnerability as they are at higher risk of neglecting alarming symptoms or being misinterpreted and, consequently, are at higher risk of prolonged diagnosis with a potentially more advanced disease at diagnosis.

Furthermore, Rethoré et al. advocate for implementation of annual surveillance for testicular cancer by palpation between 15 and 45 years [31]. As we confirm the risk of testicular cancer and identify age 20-40 as the time when almost all testicular cancer cases occur, we hope for decision makers to assess the need for testicular screening in this group. Furthermore, the 2.5% absolute risk of developing leukaemia during childhood underscores the impact of good management and treatment of leukaemia in this group. However, as acute leukaemias often have rapid onset, previous data have shown limited benefit of surveillance or routine testing for early detection [33].

The potential benefits from understanding genetic underlying mechanisms to the unique cancer associations seen in Down syndrome could be vast, with applications not only in individuals with Down syndrome, but also in individuals with cancer overall. Consequently, the results presented in this study necessitate further investigation of the role of trisomy 21 on carcinogenesis.

## Conclusion

This nationwide cohort study provides the most comprehensive profile to date of subtype- and age-specific cancer risks in individuals with Down syndrome. Our results, together with previous studies, support an increased risk of leukaemia during childhood, and an increased risk of testicular cancer, and possibly liver and gallbladder tumours, and chondroblastoma/chondrosarcomas in adults. The cancer risk was significantly decreased for major tumour types including breast, prostate, lung, colorectal, gynaecological cancers, and melanomas, and a lower risk was indicated for several other cancer types. Defining the cancer profile is essential to inform clinical decisions on surveillance and early detection. Given our results, we encourage evaluation of risk-benefit to follow the general adult routine screening programs and implementation of testicular cancer screening. Ultimately, we call for a consensus document on surveillance strategies in individuals with Down syndrome. Furthermore, the results importantly implicate the role of chromosome 21 in oncogenesis and warrant future studies on the topic.

## Supplementary information

Supplementary information

STROBE checklist

## Data Availability

Pseudonymized personal data were obtained from the national Swedish Registry holders after ethical approval and secrecy assessment. According to Swedish laws and regulations, personal sensitive data can only be made available to researchers who fulfil legal requirements for access. Contact Associate Professor Ann Nordgren (ann.nordgren@ki.se) for questions about data access.
