# Supplementary material for: Lifetime risk of solid tumors and leukemia in Down Syndrome: a population-based Swedish matched cohort study
Source: Br J Cancer. 2025 Dec 26;134(5):790–8. doi: 10.1038/s41416-025-03318-5 (PMC12905194; doi:10.1038/s41416-025-03318-5)
Supplement: Supplementary file 1 — Supplementary information [file 41416_2025_3318_MOESM1_ESM.pdf]

Supplementary Materials

	Page no.
**Figure S1. Cohort flow chart.**	2
**Table S1. List of ICD codes used for identification of individuals with Down Syndrome.**	3
**Table S2. Estimates of leukemia risk in individuals with Down Syndrome, stratified by age, gender and subgroups of leukemia.** Full list of estimates in various age intervals for leukemia and subgroups of leukemia.	4
**Table S3. Sensitivity analysis of solid tumor risk in individuals with DS, restricted to individuals aged 20-60**.	5
**Table S4. Sensitivity analysis of cancer risk in individuals with Down Syndrome, only including individuals diagnosed with Down Syndrome on two or more occasions.**	6
**Table S5. Sensitivity analysis investigating risk of solid tumors (including lymphomas) and leukemia in children with Down syndrome born between 1973-2017, stratified by age and cancer type.**	7
**Table S6. Sensitivity analysis investigating risk of adult solid tumors (including lymphomas) in individuals with Down syndrome born between 1950-2017, stratified by age and sex.**	8
**Table S7. Sensitivity analysis investigating risk of adult solid tumors (including lymphomas) in individuals with Down syndrome born between 1938-2017, stratified by age and sex.**	9

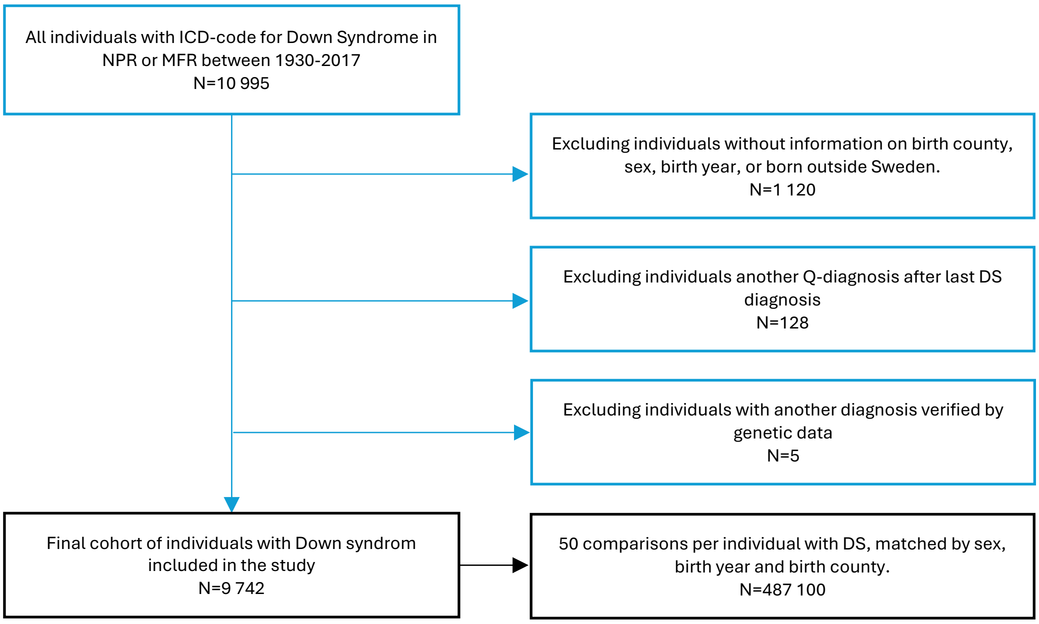

Figure S1. Flow chart of study participants.

Table S1. ICD codes used for identification of individuals with Down Syndrome.

	ICD code	Explanation
**ICD-7**	325,4	Mongolismus
**ICD-8**	759,3	Trisomia 21-22, morbus Down, mongolismus
	31050	Retardatio mentalis, lindrigt under normal intelligens, Morbus Down (mongolism), ej kromosomundersökt
	31051	Retardatio mentalis, lindrigt under normal intelligens, Morbus Down cum trisomia 21 sive alia abnormitas chromosomatica
	31150	Retardatio mentalis, lätt mental retardation, Morbus Down (mongolism), ej kromosomundersökt
	31151	Retardatio mentalis, lätt mental retardation, Morbus Down cum trisomia 21 sive alia abnormitas chromosomatica
	31250	Retardatio mentalis, måttlig mental retardation, Morbus Down (mongolism), ej kromosomundersökt
	31251	Retardatio mentalis, måttlig mental retardation, Morbus Down cum trisomia 21 sive alia abnormitas chromosomatica
	31350	Retardatio mentalis, svår mental retardation, Morbus Down (mongolism), ej kromosomundersökt
	31351	Retardatio mentalis, svår mental retardation, Morbus Down cum trisomia 21 sive alia abnormitas chromosomatica
	31450	Retardatio mentalis, djup mental retardation, Morbus Down (mongolism), ej kromosomundersökt
	31451	Retardatio mentalis, djup mental retardation, Morbus Down cum trisomia 21 sive alia abnormitas chromosomatica
	31550	Retardatio mentalis, mental retardation UNS, Morbus Down (mongolism), ej kromosomundersökt
	31551	Retardatio mentalis, mental retardation UNS, Morbus Down cum trisomia 21 sive alia abnormitas chromosomatica
**ICD-9**	758A	Downs syndrom, Trisomi 21 eller 22, Trisomi G
**ICD-10**	Q90	Downs syndrom

Table S2. Leukemia risk in individuals with Down Syndrome, stratified by age, gender and subgroups of leukemia.

	Down syndrome No.	Comparisons No.	Hazard Ratio (95% CI)°
**All leukemia cases**	148	951	11.08* (9.26-13-26)
Female	73	411	12.96* (10.00-16.79)
Male	75	540	9.70* (7.57-12.45)
Age at diagnosis:			
0-19	141	225	35.41 (28.02-44.74)
<5	104	119	46.43 (34.74-62.05)
5-19	37	106	22.41 (14.74-34.05)
20+	6	720	0.65 (0.29-1.45)
**Subtypes**			
**ALL**	69	207	17.88* (13.56-23.57)
Female	35	81	24.89 (16.55-37.42)
Male	34	126	13.85 (9.46-20.29)
Age at diagnosis:			
0-19	66	164	21.86 (16.06-29.77)
<5	29	90	16.68 (10.64-26.16)
5-9	18	49	22.61 (12.48-40.99)
10-19	19	25	45.09 (21.90-92.83)
20+	3	42	3.69 (1.14-11.95)
**AML**	60	207	20.00* (14.78-27.06)
Female	31	106	20.04* (13.16-30.51)
Male	29	101	19.96* (12.92-30.84)
Age at diagnosis:			
0-19	60	33	125.21* (72.42-216.47)
<5	60	11	481.24 (171.53-1350)
<1	17	3	472.81 (64.56-3462)
1-4	43	8	561.95 (150.53-2098)
5+	0	196	-
**AMKL**	13	1	-
Age at diagnosis:			
<5	13	1	-
**MDS**	<3	74	2.88* (0.69-12.07)
**Other and unspecified leukemia**	17	464	2.87* (1.76-4.69)

In analyses for adult cancer individuals were included from 1930-2017. For childhood cancer analyses (cancer at age <20) only individuals born 1958-2017 were included.

ALL= Acute Lymphoid Leukemia, AML=Acute Myeloid Leukemia, AMKL=Acute Megakaryoblastic Leukemia, MDS=Myelodysplastic syndrome

° Hazard ratios are adjusted for matching factors (birth year, sex and birth county) in all analyses. For childhood cancer cases (cancer <20 years) hazard ratios are also adjusted for maternal age, paternal age and highest educational level.

* Does not meet proportional hazard assumptions.

Table S3. Sensitivity analysis of solid tumor risk in individuals with DS restricted to age 20-60.

	20-60		
	Down Syndrome No.	Comparisons No.	Hazard Ratio (95% CI)°
Solid tumors	108	11490	0.52* (0.43-0.63)
Sex			
Female	54	6937	0.43 (0.33-0.56)
Male	54	4553	0.67 (0.51-0.88)
**Cancer site**			
Breast	16	2874	0.31 (0.19-0.50)
Prostate (age 40-60)	-	960	-
Lung (age 40-60)	<3	516	0.22 (0.06-0.90)
Digestive organs	16	1552	0.60 (0.37-0.99)
Upper gastrointestinal tract	4	294	0.74 (0.28-2.0)
Liver & Gallbladder	6	166	2.17 (0.95-4.92)
Pancreas	<3	174	0.35 (0.05-2.50)
Colorectal	5	874	0.34 (0.14-0.81)
Melanoma	9	1314	0.37 (0.19-0.72)
Skin (excl. melanoma)	<3	281	0.42 (0.10-1.69)
Gynecological	13	1244	0.59 (0.34-1.01)
Cervix	-	436	-
Ovary	5	386	0.71* (0.29-1.72)
Uterus (corpus uteri)	6	298	1.22 (0.54-2.75)
Lymphoma	11	668	0.92 (0.50-1.66)
Central nervous system	7	581	0.65 (0.31-1.38)
Head and neck	<3	342	0.32 (0.08-1.30)
Bladder or urether	3	215	0.79 (0.25-2.48)
Kidney	<3	269	0.21 (0.03-1.49)
Endocrine	<3	334	0.16 (0.02-1.16)
Testicular	27	404	3.61 (2.44-5.35)
Bone	3	58	2.69 (0.84-8.64)
Chondrosarcoma/Chondroblastoma	3	23	6.74 (2.00-22.70)

Upper gastrointestinal tract includes esophagus, stomach and small intestine. Gynecological site includes vulva, vagina, cervix uteri, ovary, uterus and other unspecified female genitalia. Hazard ratios are inherently adjusted for matching factors (birth year, sex and birth county) in all analyses. For cancer sites lung and prostate, individuals were included from age 40 (instead of 20) as these cancers mainly occur in older individuals.

Table S4. Sensitivity analysis of cancer risk in individuals with Down Syndrome, only including individuals diagnosed with Down Syndrome on two or more occasions.

	Down Syndrome No.	Comparisons No.	Hazard Ratio (95% CI)°
**Individuals in total**	8361	418 050	
**Adult malign solid tumors (age 20+)**	106	17966	0.47* (0.39-0.57)
Age at diagnosis			
20-39	39	2227	0.91 (0.66-1.25)
40-59	44	7121	0.34 (0.25-0.46)
60+	23	8618	0.44 (0.29-0.66)
Sex			
Female	48	8881	0.40 (0.30-0.53)
Male	58	9085	0.55 (0.43-0.72)
**Childhood solid tumors (age 0-19)**	7	652	0.54 (0.26-1.14)
Age at diagnosis			
<5	<3	215	0.44 (0.11-1.78)
5-19	5	437	0.60* (0.25-1.45)
Sex			
Female	<3	302	0.33 (0.08-1.34)
Male	5	350	0.73 (0.30-1.76)
Breast	18	3268	0.39 (0.24-0.62)
Prostate (40+)	<3	3191	0.03 (0.0-0.24)
Lung (40+)	0	1123	-
Digestive organs	11	2779	0.38 (0.21-0.68)
Upper gastrointestinal tract	3	484	0.51 (0.16-1.60)
Liver & Gallblader	3	281	1.04 (0.33-3.27)
Pancreas	<3	358	0.28 (0.04-2.02)
Colorectal	4	1595	0.25 (0.09-0.66)
Melanoma	7	1436	0.33 (0.16-0.69)
Skin (excl. melanoma)	5	736	0.80 (0.33-1.94)
Gynecological	8	1373	0.41 (0.20-0.81)
Cervix	0	363	-
Ovary	3	381	0.51 (0.16-1.58)
Uterus (corpus uteri)	3	478	0.59 (0.19-1.85)
Lymphoma	8	762	0.73 (0.36-1.46)
Central nervous system	3	573	0.33 (0.11-1.03)
Head and neck	3	473	0.47 (0.15-1.47)
Bladder or urether	5	423	1.20 (0.49-2.92)
Kidney	0	422	-
Endocrine	<3	319	0.19 (0.03-1.35)
Testicular	24	348	3.7 (2.44-5.60)
Bone	3	59	3.05 (0.95-9.81)
Chondrosarcoma/Chondroblastoma	3	25	7.16 (2.12-24.20)

In analyses for adult cancer individuals were included from 1930-2017. For childhood cancer analyses (cancer<20) only individuals born 1958-2017 were included. Upper gastrointestinal tract includes esophagus, stomach and small intestine. Gynecological site includes vulva, vagina, cervix uteri, ovary, uterus and other unspecified female genitalia.
°Hazard ratios are inherently adjusted for matching factors (birth year, sex and birth county) in all analyses. For childhood cancer cases (cancer <20 years) HRs are also adjusted for maternal age, paternal age and highest educational level.
* Does not meet proportional hazard assumptions.

Table S5. Sensitivity analysis investigating risk of solid tumors (including lymphomas) and leukemia in children with Down syndrome born between 1973-2017, stratified by age and cancer type.

	Down Syndrome No.	Comparison No.	Hazard ratio (95% CI)°
**Individuals in total**	5 977	298 850	
**Solid tumors (0-19 y)**	10	573	0.93 (0.49-1.74)
Age at diagnosis			
<5	5	196	1.27 (0.52-3.11)
5-19	5	377	0.74* (0.30-1.79)
Sex¤			
Female	3	266	0.62 (0.20-1.93)
Male	7	307	1.18 (0.56-2.52)
**Leukemia (0-19 y)**	133	183	41.03 (32.01-52.59)
ALL (0-19 y)	62	141	23.80 (17.23-32.86)
<5	27	80	17.40 (10.88-27.83)
5-9	17	43	25.04 (13.47-46.54)
10-19	18	18	51.95 (23.79-113.47)
AML (0-19 y)	60	26	180.74* (94.85-344-42)
<5	60	10	498.97 (176.81-1408.17)

°Hazard ratios are inherently adjusted for matching factors (birth year, sex and birth county) in all analyses. For childhood cancer cases (cancer <20 years) HRs are also adjusted for maternal age, paternal age and highest educational level.

* Does not meet proportional hazard assumptions.

Table S6. Sensitivity analysis investigating risk of adult solid tumors (including lymphomas) in individuals with Down syndrome born between 1950-2017, stratified by age and sex.

	Down Syndrome No.	Comparisons No.	Hazard ratio (95% CI)°
**Individuals in total**	8457	422 850	
**Adult solid tumors (20+ y)**	91	8 971	0.61* (0.50-0.75)
Age at diagnosis			
20-39	40	2209	0.99 (0.72-1.35)
40-59	45	5307	0.48 (0.35-0.64)
60+	6	1455	0.44 (0.20-1.00)
Sex			
Female	45	4 917	0.53 (0.40-0.72)
Male	46	4 054	0.71 (0.53-0.96)

°Hazard ratios are inherently adjusted for matching factors (birth year, sex and birth county) in all analyses.

* Does not meet proportional hazard assumptions.

Table S7. Sensitivity analysis investigating risk of adult solid tumors (including lymphomas) in individuals with Down syndrome born between 1938-2017, stratified by age and sex.

	Down Syndrome No.	Comparisons No.	Hazard ratio (95% CI)°
**Individuals in total**	9473	473 650	
**Adult solid tumors (20+ y)**	133	19 840	0.51* (0.43-0.61)
Age at diagnosis			
20-39	47	2704	0.93 (0.70-1.24)
40-59	62	8525	0.41 (0.32-0.53)
60+	24	8611	0.41 (0.28-0.62)
Sex			
Female	68	10 127	0.48 (0.38-0.61)
Male	65	9713	0.55 (0.43-0.70)

°Hazard ratios are inherently adjusted for matching factors (birth year, sex and birth county) in all analyses.

* Does not meet proportional hazard assumptions.
